# Surgical Outcomes of Stanford Type A Aortic Dissection in Jehovah’s Witness Patients

**DOI:** 10.5761/atcs.oa.25-00124

**Published:** 2026-01-09

**Authors:** Kokoro Tabata, Kosaku Nishigawa, Motoharu Shimozawa, Shunya Ono, Takeyuki Kanemura

**Affiliations:** Department of Cardiovascular Surgery, IMS Katsushika Heart Center, Tokyo, Japan

**Keywords:** Jehovah’s Witness, Stanford type A aortic dissection, bloodless surgery, total arch replacement, hemiarch replacement

## Abstract

**Purpose:**

Surgery for Jehovah’s Witness patients with Stanford type A aortic dissection (TAAD) carries a high surgical risk, and few reports have examined outcomes in this patient population. This study evaluated perioperative outcomes of surgery for TAAD in Jehovah’s Witness patients.

**Methods:**

Eight Jehovah’s Witness patients who underwent surgery for TAAD at our institution between February 2016 and January 2025 were retrospectively reviewed. No patients were receiving antiplatelet or anticoagulant therapy at the time of surgery. Preoperative characteristics, operative data, and postoperative outcomes were assessed.

**Results:**

Emergency ascending aortic replacement was performed in 6 patients, while the 2 patients who underwent elective surgery due to a chronic course or thrombosed false lumen received total or partial arch replacement. Both elective cases received preoperative iron supplementation. The median postoperative nadir hemoglobin was 9.2 (interquartile range, 6.8–9.6) g/dL. One patient died, one was transferred to rehabilitation, and 6 patients (75.0%) were discharged home without major complications.

**Conclusions:**

Perioperative outcomes of surgery for TAAD in Jehovah’s Witness patients were favorable. Proper surgical timing and preoperative management are essential to achieving satisfactory results. Further investigation with a larger cohort and longer follow-up is warranted.

## Introduction

Cardiovascular surgery often entails significant blood loss. Patients with Stanford type A aortic dissection (TAAD) may present with coagulopathy and frequently require circulatory arrest (CA) during surgery, increasing the risk of bleeding.^1,2)^ Preoperative anemia, often resulting from aortic rupture or a thrombosed false lumen, is common. Anemia, both preoperative and postoperative, has been associated with adverse outcomes and often necessitates transfusion.^3,4)^ However, Jehovah’s Witness patients refuse transfusion, leading many cardiac surgeons to hesitate in performing surgery, particularly in high-risk cases such as TAAD. Consequently, only a few reports have described surgical outcomes in this population. This study reports the perioperative outcomes of surgery for TAAD in Jehovah’s Witness patients treated at our institution.

## Patients and Methods

### Patient population

Among 430 patients who underwent surgery for TAAD between February 2016 and January 2025 at IMS Katsushika Heart Center, Tokyo, Japan, 8 were Jehovah’s Witnesses. **Table 1** summarizes patient characteristics. The median age was 72 (interquartile range [IQR], 68–76) years, and all patients were independent in activities of daily living preoperatively. Two patients (Cases 2 and 3) had cardiac tamponade, and Case 2 experienced cardiac arrest prior to surgery. Case 7 had significant aortic regurgitation (AR) requiring intervention. Emergency surgery was performed in 6 patients; Cases 6 and 7 underwent elective surgery. Although Case 6 had an acute presentation, the dissection involved a thrombosed false lumen type and recent coronary stenting, and the patient was receiving dual antiplatelet therapy; surgery was delayed to mitigate bleeding risk. Case 7 was diagnosed incidentally by computed tomography, presented without symptoms, and was suspected to be chronic; elective surgery was selected. Both elective patients received preoperative erythropoietin, and Case 7 also received iron supplementation. The median preoperative hemoglobin level was 13.1 (IQR, 11.7–14.3) g/dL, and the platelet count was 16.3 × 10^4^ (IQR, 12.7–18.5)/µL. The median preoperative D-dimer level was 28.2 (IQR, 3.7–77.6) µg/mL, and the median fibrinogen level was 146 (IQR, 120–276) mg/dL. No patient was receiving antiplatelet or anticoagulant therapy at the time of surgery. All patients provided informed consent for surgery after a thorough explanation of the surgical risks, in accordance with the Japanese guidelines on religious refusal of blood transfusion.^5)^

**Table 1:** Preoperative patient characteristics

Case	Diagnosis	False lumen	Comorbidities	Urgency	Age (years)	Sex	BSA (cm^2^)	Hb (g/dL)	Plt (×10^4^/µL)	Alb (g/dL)	D-dimer (µg/mL)	Fib (mg/dL)
1	ATAAD	Patent	None	Emergency	67	Female	1.65	11.6	8.2	3.7	33.3	122
2	ATAAD	Patent	Cardiac tamponade	Emergency	78	Female	1.38	11.5	12.1	3.2	180.5	38
3	ATAAD	Thrombosed	Cardiac tamponade	Emergency	68	Male	1.79	13.5	18.6	4.0	60.0	152
4	ATAAD	Thrombosed	None	Emergency	73	Female	1.65	15.3	18.4	4.5	3.4	272
5	ATAAD	Patent	None	Emergency	45	Male	2.07	14.9	14.5	4.1	23.1	118
6	ATAAD	Thrombosed	TAA	Elective	71	Male	2.01	11.8	13.2	2.8	2.1	304
7	CTAAD	Patent	AR	Elective	80	Female	1.49	13.6	36.8	4.2	4.0	279
8	ATAAD	Patent	None	Emergency	73	Female	1.6	12.7	18.1	3.8	95.1	139

Alb: albumin; AR: aortic regurgitation; ATAAD: acute type A aortic dissection; BSA: body surface area; CTAAD: chronic type A aortic dissection; Fib: fibrinogen; Hb: hemoglobin; Plt: platelet count; TAA: thoracic aortic aneurysm

Operative and postoperative data and outcomes were retrospectively evaluated for all 8 Jehovah’s Witness patients who underwent surgery for TAAD.

### Surgical techniques

All patients received central venous, bilateral radial, and right dorsal pedal arterial lines preoperatively. Surgery was performed via median sternotomy. Cardiopulmonary bypass (CPB) was established by cannulation of the left femoral artery for arterial return and the right atrium for venous drainage. A left ventricular vent was placed via the right upper pulmonary vein. Except in cases of rupture involving the ascending aorta or aortic root, clamping of the ascending aorta was generally avoided. CA was initiated after confirming that the rectal or bladder temperature had decreased below 28°C. Myocardial protection was achieved using retrograde cold blood cardioplegia, with selective antegrade delivery added as needed. For total arch replacement (TAR) and partial arch replacement (PAR), retrograde cerebral perfusion (RCP) was initiated at the onset of CA, followed by selective cerebral perfusion (SCP) after cannulation of the 3 cervical branches. In cases undergoing hemiarch replacement (HAR), RCP was continued until reperfusion. Triplex (Vascutek, Terumo, Tokyo, Japan) or J-Graft (Japan Lifeline, Tokyo, Japan) prostheses were used, with distal anastomosis performed using the turn-up technique.^6)^ Reperfusion was initiated via a side branch of the graft following the distal anastomosis. The cervical branches were reconstructed in the order of left subclavian, left common carotid, and brachiocephalic arteries. Proximal anastomosis was performed at the level of the sinotubular junction using internal and external felt strips.

Although resection of the primary entry site with prosthetic graft replacement is standard practice at our institution, procedures were individualized in Jehovah’s Witness patients due to transfusion constraints.

### Statistical analysis

Descriptive statistics were used to summarize patient characteristics and outcomes. Continuous variables are reported as mean ± standard deviation or median with IQR, depending on their normality. No comparative or inferential statistical analyses were performed due to the small sample size.

## Results

### Operative data

**Table 2** summarizes the intraoperative data. All 6 emergency surgery cases underwent HAR. Case 6 (elective) received TAR due to a distal arch aneurysm, and Case 7 (elective) underwent PAR with aortic valve replacement for AR. The median operative time, CPB time, CA time, and aortic cross-clamp time were 316 (IQR, 268–392), 161 (IQR, 117–219), 40 (IQR, 32–57), and 108 (IQR, 88–140) min, respectively. The median intraoperative blood loss was 1704 (IQR, 1271–2509) mL. At the time of CA, the pharyngeal and bladder temperatures were 24.4 (IQR, 22.4–26.3)°C and 27.2 (IQR, 25.3–27.7)°C, respectively. No allogeneic transfusions were administered in any patient.

**Table 2:** Intraoperative data

Case	Surgical procedure	Aortic cross-clamp	Cerebral perfusion	CPB time (min)	Circulatory arrest time (min)	Cardiac arrest time (min)	Operation time (min)	Pharyngeal temperature (°C)	Bladder temperature (°C)	Autologous blood reinfusion (mL)	ANH (mL)	Intraoperative blood loss (mL)
1	HAR	–	RCP	114	33	84	265	27.6	27.8	331	0	1374
2	HAR	+	RCP	119	31	91	385	24.8	24.8	2794	0	4018
3	HAR	–	RCP	99	23	60	175	27.1	27.5	240	0	928
4	HAR	–	RCP	168	52	112	275	23.9	27.1	522	1200	1522
5	HAR	–	RCP+SCP	215	62	113	405	20.7	28.1	243	1600	1195
6	TAR	–	RCP+SCP	254	69	185	399	22.8	27.2	502	800	2180
7	PAR+AVR	–	RCP+SCP	222	44	167	358	25.4	25.7	482	0	1886
8	HAR	–	RCP	154	36	103	271	22.0	24.8	574	0	2838

ANH: acute normovolemic hemodilution; AVR: aortic valve replacement; CPB: cardiopulmonary bypass; HAR: hemiarch replacement; PAR: partial arch replacement; RCP: retrograde cerebral perfusion; SCP: selective cerebral perfusion; TAR: total arch replacement

### Postoperative data

Postoperative data are summarized in **Table 3**. The median nadir hemoglobin level was 9.2 (IQR, 6.8–9.6) g/dL, and the median hemoglobin level at discharge was 10.5 (IQR, 9.9–11.5) g/dL. The median platelet count and fibrinogen level measured immediately after surgery were 9.8 ×10^4^ (IQR, 4.1–10.9)/µL and 146 (IQR, 122–178) mg/dL, respectively. No cases required reoperation for bleeding. The median 24-h chest drain output was 390 (IQR, 305–450) mL. Median intensive care unit (ICU) and hospital stays were 5 (IQR, 5–8) and 16 (IQR, 11–26) days, respectively. All patients except Cases 4 and 7 received postoperative iron supplementation; Cases 2 and 5 also received erythropoietin therapy. In some cases, with the patient’s consent, blood fraction products were administered perioperatively as needed. Albumin products were administered intraoperatively and postoperatively in Cases 2 and 5. Fibrinogen was administered intraoperatively in Case 5 and postoperatively in Case 2.

**Table 3:** Postoperative data

Case	Nadir Hb (g/dL)	Hb at discharge (g/dL)	Plt, POD 0 (×10^4^/µL)	Fib, POD 0 (mg/dL)	Chest drain output at 24 h (mL)	Complications	Outcome	Hospital stay (days)	ICU stay (days)	Iron supplementation	ESA supplementation
1	9.3	12.0	4.7	NA	410	None	Discharged home	11	3	Postoperative	None
2	3.9	7.3	3.2	ND	910	CI, AF, MOF	Dead	5	5	Postoperative	Postoperative
3	9.6	9.6	9.4	90	490	None	Discharged home	17	4	Postoperative	None
4	10.6	11.7	10.2	156	240	AF	Discharged home	14	5	None	None
5	5.3	11.2	11.2	135	410	CI, AF, RF, UTI	Transferred to another facility	122	23	Postoperative	Postoperative
6	8.3	10.7	3.4	416	300	Pneumonia	Discharged home	33	9	Postoperative	Preoperative
7	9.5	10.2	11.5	178	370	None	Discharged home	10	6	Preoperative	Preoperative
8	9.0	10.2	10.5	122	310	None	Discharged home	18	5	Postoperative	None

AF: atrial fibrillation; CI: cerebral infarction; ESA: erythropoiesis-stimulating agent; Fib: fibrinogen; Hb: hemoglobin; ICU: intensive care unit; MOF: multiple organ failure; NA: not available; ND: not detectable; POD: postoperative day; Plt: platelet count; RF: renal failure; UTI: urinary tract infection

Postoperative complications included symptomatic stroke in 2 patients (25%), renal failure requiring dialysis in 2 (25%), multi-organ failure in 1 (12.5%), atrial fibrillation in 3 (37.5%), pneumonia in 1 (12.5%), and urinary tract infection in 1 (12.5%). Case 2 died of multi-organ failure on postoperative day 34, resulting in an operative mortality of 12.5% (1/8). Case 5, who experienced a symptomatic stroke, underwent tracheostomy and was transferred to rehabilitation on postoperative day 122. Six patients (75%) were discharged home.

## Discussion

As of 2024, the worldwide population of Jehovah’s Witnesses is estimated at approximately 9.04 million, including about 210000 in Japan as of 2023. Although relatively small compared with the general population, this number is clinically significant. Jehovah’s Witnesses commonly refuse blood transfusions on the basis of their religious beliefs. In Japan, the right of competent adults to refuse transfusion is legally recognized as an aspect of personal autonomy. Transfusion refusal can be broadly classified into 2 categories: absolute refusal, in which transfusion is declined under any circumstances, and relative refusal, in which exceptions may be permitted depending on the situation. The acceptance of CPB and blood fraction products is left to individual discretion. At our institution, all Jehovah’s Witness patients who underwent cardiovascular surgery adopted a position of relative refusal of blood transfusion. In these cases, plasma derivatives and other blood fraction products were administered only within the limits of preoperatively obtained consent, while CPB and cell salvage were used in all cases with patient agreement. This approach allowed us to respect the patients’ religious beliefs while providing perioperative management consistent with current clinical practice.

The present study demonstrated perioperative outcomes of 8 Jehovah’s Witness patients who underwent surgery for TAAD. Despite the inability to use allogeneic blood transfusions and the urgency of most cases, outcomes were generally favorable, with an operative mortality of 12.5% and 6 patients (75%) discharged home. None of the discharged patients experienced major complications such as stroke or renal failure. Reports on Jehovah’s Witness patients undergoing surgery for TAAD remain limited. A discussion of the perioperative management strategies used in this cohort may contribute to improving perioperative outcomes in this challenging patient population.

Previous studies have reported no significant differences in outcomes between Jehovah’s Witness and non-Witness patients undergoing cardiac surgery.^7,8)^ However, TAAD surgeries involve greater bleeding risks compared with routine cardiac procedures. Data on Jehovah’s Witness patients undergoing TAAD surgery remain limited, with only a few case reports and a 6-case series published by West et al.^9)^ In Japan, national in-hospital mortality rates for TAAD are approximately 10%,^10,11)^ suggesting that the outcomes observed in the present study were acceptable. Preoperative and postoperative anemia management, appropriate surgical timing, and careful procedure selection likely contributed to the favorable outcomes.

Multiple studies have reported an association between preoperative anemia and surgical outcomes.^3,4)^ In a report by Tanaka et al., preoperative hemoglobin level was identified as an independent predictor of in-hospital mortality and postoperative complications in Jehovah’s Witness patients undergoing cardiac surgery.^12)^ These findings highlight the importance of correcting anemia prior to surgery in this population. Although emergency surgery is the standard approach for acute TAAD (ATAAD) due to its rapidly worsening prognosis,^13)^ several studies have documented favorable outcomes following initial conservative management in patients with false lumen-thrombosed–type aortic dissection.^14,15)^ Elective surgery with preoperative correction of anemia may be an appropriate strategy in cases of false lumen-thrombosed and chronic aortic dissection. In the present series, 2 patients underwent elective repair with preoperative erythropoiesis-stimulating agents and were discharged without major complications.

In Jehovah’s Witness patients, the inability to receive allogeneic blood transfusions necessitates alternative strategies compared with standard management of TAAD. In Japan, no significant differences in in-hospital mortality or stroke incidence have been observed between HAR and TAR for ATAAD.^16)^ TAR has been reported to reduce long-term risk of reoperation compared with HAR, making it a reasonable choice for younger patients due to favorable early outcomes.^17)^ However, because TAR is associated with a higher risk of massive intraoperative bleeding, HAR may provide better early outcomes in Jehovah’s Witness patients who refuse blood transfusions.^18)^ At our institution, HAR is selected as the primary surgical option for Jehovah’s Witness patients with TAAD, and extensive surgical interventions or additional specific procedures are considered based on anatomical and clinical factors.

Postoperative hemoglobin levels have also been associated with surgical outcomes. Zhou et al. reported that, among patients undergoing cardiac surgery with CPB, those with lower postoperative nadir hemoglobin levels had higher rates of complications and mortality compared with those with higher levels.^19)^ Murphy et al. found higher mortality rates in patients transfused at hemoglobin levels <7.5 g/dL than in those transfused at <9.0 g/dL.^20)^ In the present series, one fatality occurred in a patient with a nadir hemoglobin of 3.9 g/dL, and another patient with a level of 5.3 g/dL presented with stroke and renal failure. At our institution, intraoperative blood suction during CPB is performed using a pump sucker instead of a cell saver to minimize blood loss. Postoperatively, iron supplementation was administered in 6 cases to correct anemia, and erythropoiesis-stimulating agents were used in selected patients. Because blood sampling in the ICU has been reported to contribute to postoperative anemia, arterial blood gas analysis is typically performed every 3 h after surgery; however, in Jehovah’s Witness patients, this is limited to clinically indicated situations, and other blood tests are also minimized.^21)^

### Study limitations

This study has several limitations. First, it was a single-center retrospective study involving a small cohort. Second, follow-up durations varied among patients, limiting the assessment of long-term outcomes. Third, variations in surgical techniques and perioperative management among surgeons may have influenced the results.

## Conclusions

Thoracic aortic surgery for TAAD in Jehovah’s Witness patients can yield acceptable perioperative outcomes without the use of allogeneic blood transfusion, provided that careful preoperative planning and surgical timing are employed. Further investigation with larger cohorts and long-term follow-up is warranted.

## Declarations

### Data availability

The data that support the findings of this study are available from the corresponding author upon reasonable request.
